# Seeking out the sweet spot in cancer therapeutics: an interview with Lewis Cantley

**DOI:** 10.1242/dmm.026856

**Published:** 2016-09-01

## Abstract

Lewis C. Cantley, Director of the Sandra and Edward Meyer Cancer Center at Weill Cornell Medicine, is a world leader in cancer and metabolic disease research. His seminal discoveries have shed light on the regulation of ion pumps and other transport proteins, insulin-mediated regulation of glucose metabolism and the role of signal transduction networks in cell transformation. At Tufts University in the 1980s, Lewis and his collaborators unveiled and characterized the phosphoinositide 3-kinase (PI3K) signaling pathway; a discovery that revolutionized the field of lipid signaling. In this interview, he documents his journey from serendipitous discovery of the pathway to determining its diverse physiological functions and role in cancer – an incredible odyssey that has laid the groundwork for clinical trials based on PI3K inhibitors. He also discusses the impact his early life had in spurring a thirst to understand biological processes at the molecular level, highlights how his multiple collaborations have helped in translating his basic discoveries to the clinic and explains why eating a high-sugar diet can be harmful. Ongoing studies in the Cantley lab are aimed at determining the mechanistic underpinnings of pancreatic, colorectal, ovarian and breast cancers, particularly the role of cellular metabolic pathways. The group has recently shown, amongst other breakthroughs, that vitamin C could provide a promising therapy for certain hard-to-treat cancers.

## Abstract (precis)

**Summary:** We spoke to Lewis Cantley about his career path and the story behind some of his key breakthroughs, including discovery of the phosphoinositide 3-kinase signaling pathway and insights into the role of dysregulated metabolism in cancer.


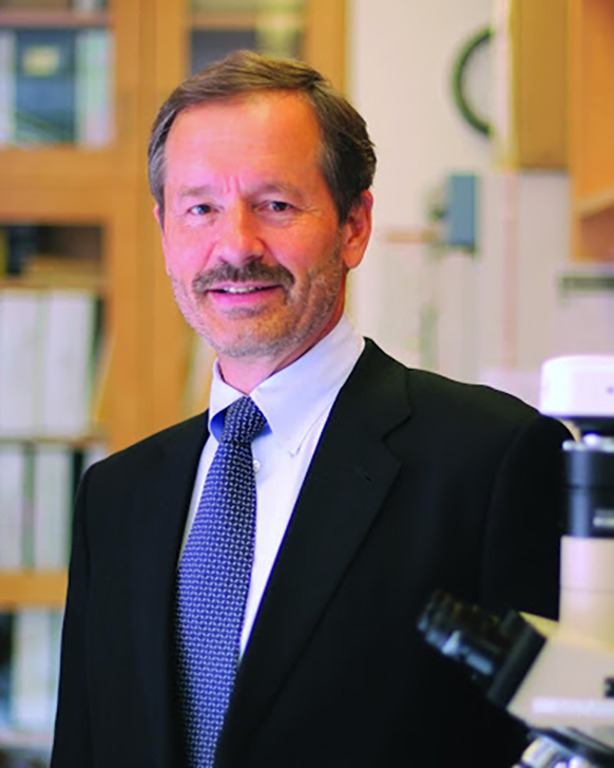


**I once read that, as a child, you worked out how to build fireworks. Did you always know that you would end up as a scientist?**

Firecrackers were illegal where I grew up in West Virginia. When I was around 10-11 years old, I wanted to buy some, so my dad said “why don't you make your own?” He wasn't a chemist, but he was a very smart man and he figured out from reading the encyclopedia that we needed just three ingredients. I went to the drug store, bought the ingredients and made my own gunpowder. It wasn't very effective in blowing up, but it burned very well. I started using my homemade gunpowder as a fuel to try to launch rockets – and it worked, although I didn't get them very high. Today I would probably be arrested based on the buying pattern I had at the local drug store!

I did know I wanted to be a scientist from very early on. I was largely influenced by my father who, whenever I asked a question about how things work, would always come up with a logical explanation rather than resort to saying “because God made it that way”. If he didn't know the answer, he would refer to the encyclopedia. By the time I started school I realized that I actually knew a lot more science than most of my teachers. I had a talent for it, particularly chemistry. One of my presents growing up was a chemistry set, and I loved mixing things together and seeing colors change and causing occasional explosions. I just found that amazingly fun.

**When did you start using your talent for chemistry to answer biological questions?**

I was always interested in biology, too. I grew up on a farm and loved growing tomato plants, which made me wonder how photosynthesis works. I found it a magical thing – that the sun shines on a plant and it takes off growing. I thought if we could figure out how this works, light energy could be captured to do all kinds of things. But I found high school biology very boring – we memorized a whole lot of things without getting any real mechanistic insight into the processes. I swore at that time that I would never take another course in biology. I decided it would be much better to learn chemistry and, ultimately, I suspected that there would be nothing in biology that couldn't be explained by chemistry. I liked to understand kinetics and how reactions happen, so I decided to go into biophysical chemistry. In the end, that was the right path to take, although had you told me when I was 25 years old that I would be the director of a cancer center I would have been incredulous, given that I was totally into chemistry.

“…had you told me when I was 25 years old that I would be the director of a cancer center I would have been incredulous, given that I was totally into chemistry”

**What were the major aims and findings of your PhD in Gordon Hammes's lab at Cornell University?**

I originally worked on the F_1_ portion of the ATP synthase in chloroplasts and mitochondria. At that time, the chemiosmotic hypothesis – proposed by Peter Mitchell in the early 60s – was very controversial. People were beginning to believe it but there were still a lot of holdouts; major luminaries in the field who just would not accept the concept that you could use an electrochemical gradient to synthesize a molecule. Although Mitchell's hypothesis was controversial, I found it really compelling when I was an undergraduate. That's why it was exciting for me to work on the enzyme that meditates the chemiosmotic effect as a graduate student.

**There seems to have been resurgence of interest recently in exploring mitochondrial function, particularly to understand metabolic reprogramming in disease states. How important is it to look back at the early seminal papers in this field?**

Back in the mid-80s and even into the 90s, the people who worked in the fields of ATP synthesis, the electron transport system or on mitochondrial function generally, were known – somewhat disparagingly – as the ‘mitochondriacs'. For most biochemists and biologists, these mitochondriacs were people you didn't want to be in the same room with. They spoke a different language and were working on obscure biophysical questions; membrane potentials and rates of flux and that sort of thing. Then this area of research became very unpopular. It was almost impossible to get funding to work on mitochondria, as though it was all understood and there was nothing more to do. Now suddenly there is this resurgence, and mainline biologists are getting excited about mitochondria. A lot of questions that were originally discussed in the 70s – such as the role of reverse chemiosmosis, which was documented by Peter Mitchell – are now being considered again. The mitochondriacs have been revived!

“Now suddenly there is this resurgence, and mainline biologists are getting excited about mitochondria. … The mitochondriacs have been revived!”

**Can you tell us about the path that led you to the discovery of PI3Ks?**

It was a rather convoluted pathway. In fact, I never published the series of observations that led me to start looking at phosphoinositide kinases (PI-kinases). As a post-doc at Harvard, I was reconstituting purified Na+/K+ ATPase into a synthetic lipid vesicle and adding in lipids to try to mimic what you would find in the plasma membrane of the cell. It turns out that you had to include phosphatidylinositol (PI) in the synthetic membranes in order for Na +/K+ ATPase to be fully functional. It occurred to me to ask what would happen if phosphorylated PI was used instead. So I bought some phosphatidylinositol 4-phosphate (PI4P) and phosphatidylinositol (4,5)-bisphosphate [PI(4,5)P_2_] and compared the effects of reconstituting the enzyme with the different forms of PI, and I found that the enzymatic activity changed depending on the state of the phosphorylation of the lipid.

At that time, tyrosine kinases hadn't yet been discovered, and the insulin receptor hadn't been purified – nobody knew what the latter did. I was working on how insulin activates Na+/K+ ATPase, and it occurred to me that maybe insulin stimulates phosphorylation of PI and that this would change the lipid annulus around the enzyme and regulate its activity. The idea that transport proteins like Na+/K+ ATPase could be affected by the phosphorylation state of the lipid in which it was embedded was attractive to me, and stimulated me to try to purify a PI-kinase. I wanted to find out whether adding this enzyme to Na+/K+ ATPase that had been reconstituted into a synthetic membrane containing PI would enhance the ability of Na+/K+ ATPase to transport Na+ and K+ across the membrane. If so, this would provide a possible mechanism to explain how growth factors and hormones regulate the transport of cations into and out of cells. That was my thinking as I was establishing my laboratory in the late 1970s and early 1980s. However, no PI-kinase had been purified at that time.

I then came across a paper by Ray Erikson reporting on the activity of a glycerol kinase that co-purified with the v-Src tyrosine kinases. This was in 1983, and tyrosine kinase activities were being identified in many growth factor receptors and oncogenes. I noticed that the *K*_m_ for glycerol was extremely high (∼1 M), suggesting that the true physiological substrate was probably not glycerol but a related molecule. I started wondering whether the inositol ring of PI, which is like two glycerol molecules glued together, could be the real substrate. At that time, a former post-doc from my lab, Ian Macara, was visiting and he, I and Malcolm Whitman, a graduate student, became sufficiently enthusiastic about the possibility that v-Src was driving PI phosphorylation that we decided to pursue this idea. Malcolm collaborated with a postdoctoral fellow in Ray Erikson's lab, Yoshikazu Sugimoto, and they showed that PI was a much better substrate than glycerol for the kinase activity in the v-Src preparation. Ian Macara made a similar finding using another oncogenic tyrosine kinase preparation from avian sarcoma virus (UR2). In these original papers, we made two assumptions that turned out to be incorrect. First, we thought the same catalytic activity that was phosphorylating tyrosine residues was also phosphorylating PI because temperature-sensitive mutants that lost the tyrosine kinase activity also lost the PI-kinase activity. We later learned that this was because the PI-kinase could only bind to the tyrosine kinase when the kinase was activated.

The second incorrect assumption was that the product of this PI-kinase was PI4P. At that time in the mid-1980s, PI4P was the only monophosphorylated form of PI known to exist in nature. But a couple of years later, when we purified and further characterized the PI-kinase that co-purified with v-Src (and other oncoproteins), we noticed that the monophosphorylated form of PI that it produced migrated slightly more slowly than PI4P upon thin layer chromatography analysis; literally about 1 mm difference. In collaboration with Peter Downes, we showed that the product was actually phosphorylated on the 3-hydroxyl (rather than 4-hydroxyl) position of the inositol ring [thereby identifying phosphoinositide 3-kinases (PI3Ks)]. We ultimately went on to show that the same activity could convert PI4P to phosphatidylinositol (3,4)-bisphosphate [PI(3,4)P_2_] and convert PI(4,5)P_2_ to phosphatidylinositol (3,4,5)-trisphosphate (PIP_3_). We now know that seven phosphorylated forms of PI exist, not two as originally believed. The five additional phosphoinositides were missed because they are far less abundant than PI4P and PI(4,5)P_2_. All seven of the phosphoinositides play crucial roles in cell signaling and in intracellular membrane trafficking.

**How did you come to realize that PI3K activity has a role in oncogenic transformation?**

We began collaborating with Tom Roberts' lab and Brian Schaffhausen's lab to ask how well the transforming ability of the oncoprotein polyomavirus middle T-antigen correlated with the associated Src tyrosine kinase activity and the associated PI3K activity. Malcolm from my lab formed a close collaboration with David Kaplan in Tom's lab to assess a whole series of mutants of the polyoma protein, and interestingly, we found that the mutants that could bind to Src but couldn't activate PI3K didn't transform cells. This indicated that the ability to bind to and to activate cellular PI3K was a crucial factor in transformation of cells by polyomavirus.

At that time, we were defining transformation, in part, as the ability to grow beyond confluence. The shape of the cell literally changed when you transformed them; the actin was remodeled and you could visualize membrane ruffling using time-lapse microscopy. We showed that it was a single tyrosine residue at position 315 in the polyoma protein that was directly binding to PI3K, and mutation at this site eliminated its ability to remodel the actin cytoskeleton. Other things still happened, but this particular transformation – to grow beyond confluence – was definitely lost, and this correlated with the ability to produce PIP_3_ in the cell. Immortalized cell lines make PIP_3_ when they are at low density but stop making it when they reach a high density. By contrast, cells that have been transformed with polyomavirus middle-T antigen keep making PIP_3_ when they reach a high density and just keep growing. So, confluence-dependent inhibition of PIP_3_ production could be circumvented by activating PI3K. We had figured this out by the early 90s, but we still didn't fully know how it worked.

**There was some initial skepticism in the field about the activity and oncogenic role of PI3K. Did you ever feel discouraged by this or was your belief unwavering?**

First of all, my belief was totally unwavering. Multiple members of my lab, Tom's lab and Brian's lab could see a 100-fold change in the PI-kinase activity associated with v-Src or with polyomavirus middle-T antigen upon mutating these oncoproteins so I knew it had to be true. Importantly, there was no doubt that the product of this enzyme was phosphatidylinositol 3-phosphate, not PI4P. That's the beauty of chemistry – it is very unambiguous. But people who had worked on phosphoinositides for their entire careers could not believe that phosphorylation at the 3-position occurred in nature and that they had missed this. The problem is that the phosphoinositides that we had discovered were far less abundant than PI4P and PI(4,5)P_2_, and were not easily separated from these species. People also thought that a lipid molecule with such low abundance can't possibly be important in the membrane – in fact, I had a lot of trouble publishing papers because of this. The reviewers didn't understand the concept that these lipids could act as membrane-embedded signaling molecules that amplified and diversified growth factor signals by recruiting signaling enzymes such as protein kinases to the plasma membrane. The resistance came down to the fact that the entire field had missed these lipids for 30 years, so our conclusions couldn't be true.

Several other laboratories tried to reproduce the PI3K activity assay and couldn't, but they had never worked on lipids before and so didn't realize you had to sonicate the lipids in order to make a vesicle. I had learned this as a graduate student, but these other laboratories were trained in molecular biology rather than membrane biochemistry and they had no idea how to work with membranes. They published a few papers saying that our findings weren't reproducible. We eventually had to send Malcolm and David off to these other labs with a sonicator so that they could do the assay with proper vesicles. They then reproduced our results, and finally believed us. We had had no doubt that we were right, especially as three different labs – Tom's, mine and Brian's – were getting the same results, but the field didn't accept it for a long time. There was a 4-year period when I couldn't get any grants to work on PI3K, but I used funds from my other grants to continue the work.

The other problem was that when we overexpressed PI3K – even using the purified enzyme – it would not transform 3T3 cells; instead, it killed them. It worked in immortalized epithelial cells but not in fibroblasts – we still don't fully understand why. The 3T3 assay, developed in Bob Weinberg's lab, was used as the gold standard for transformation back then, so people just didn't accept that the PI3K signaling might play a role in malignant transformation. A series of papers that we published in the mid-90s eventually convinced the bulk of cancer researchers that PI3K really is relevant for driving cancers. These included the observation that PI(3,4)P_2_ and PIP_3_ directly bind to the pleckstrin homology domain of the oncogenic protein kinase AKT to mediate its activation, and the observation, in collaboration with Peter Vogt, that the avian sarcoma virus 16 oncogene he had identified encoded an activated form of PI3K. Clinical oncologists started paying attention when the tumor suppressor gene *PTEN* was shown by Jack Dixon's lab to convert PIP_3_ back to PI(4,5)P_2_, reversing the effects of PI3K. So, by the late 1990s it was clear that high levels of PIP_3_ are a key driver in cancer.

“… people who had worked on phosphoinositides for their entire careers could not believe that phosphorylation at the 3 position occurred in nature and that they had missed this”

**What made you suspect that PI3K also plays a role in metabolic regulation?**

My interest in this really came from working on the insulin receptor. As soon as we had solved the correlation with transformation, we went back to insulin. The truth is that I had speculated back in 1984 that PI3K mediates everything that insulin does – I even made a bet with one of my post-docs. It took us a while, almost 10 years, to show that this was true, and even longer to figure out how PI3K signaling cooperates with AKT to mediate insulin-driven regulation of glucose uptake. There are still missing pieces to the puzzle, and we're still working out how it all works. For example, we only recently worked out that actin remodeling contributes to glucose uptake and metabolism.

**You are notably careful about your own diet. Is this due to your research into the role of metabolism in cancer? Why exactly is sugar such a ‘bad guy’?**

This is a dangerous question to ask me because it always launches me into a long lecture on the connections between sugar, obesity, insulin resistance and cancer. For me, this insight came when we discovered that PI3K not only drives cancer growth but also mediates insulin control of metabolism. In short, eating large amounts of sugar over several years almost invariably leads to accumulation of fat in the liver, which causes insulin resistance. The state of insulin resistance is dangerous because the pancreas secretes very high levels of insulin into the serum in an attempt to bring glucose levels back to normal. These high levels of serum insulin can drive the growth of micro-cancers in a variety of tissues, especially breast and endometrial cancers that typically have high levels of the insulin receptor. The insulin receptor activates PI3K, which drives tumor cell growth and allows tumors to survive in inappropriate locations. The high consumption of sugar over the past 40 years and the connection between obesity, elevated serum insulin and oncogenesis is almost certainly responsible for the increased rates of a variety of cancers in the developed world. It is far better to prevent cancers than to attempt to cure them after they are established. So I avoid sugar whenever possible. Some day, we will view this era of massive addiction to sugar in America in the same way that we now view the period of massive addiction to tobacco. In fact, sugar might be the more dangerous addiction because obesity, diabetes, cardiovascular disease and cancers probably have a greater negative impact on our health and the US economy than smoking-related diseases.

“Some day we will view this era of massive addiction to sugar in America in the same way that we now view the period of massive addiction to tobacco”

**What is the basis of Warburg's theory of cancer, and why is it controversial? How has your work provided support for the idea?**

The Warburg effect refers to the preferential production of ATP by aerobic glycolysis instead of oxidative phosphorylation in cancer cells. Warburg hypothesized that this effect is the root driver of cancers; an idea that created a lot of controversy in Warburg's own time and certainly after his death. We now know there are some cancers that don't show the Warburg effect: although positron emission tomography (PET) imaging using the radioactive glucose analog fluorodeoxyglucose (FDG) shows that most tumors do take up glucose much better than surrounding tissue, it doesn't hold up for all tumors. I think in part, the hypothesis fell out of favor because of these exceptions to the rule. In those days, everyone treated cancer as though it is a single disease, so phenomena that weren't observed across all cancer types were considered to be irrelevant to its etiology. We now recognize the complexity of cancer and that there are multiple ways a tumor can evolve and become addicted to different pathways.

The second factor that made Warburg's theory fall out of favor was the discovery of oncogenes. Warburg was really pushing the idea that cancer is a metabolic and not a viral disease. The discovery of oncogenes suggested that cancer is actually driven by genetic aberrations. Of course, now that we know what oncogenes do, we can see that they indirectly or occasionally directly regulate metabolism. It has taken a long time – and major advances in understanding how oncogenes interact with cellular sugars – to come to realize the truth in Warburg's ideas, although many of the biochemical mechanisms are still not fully understood.

How did my work contribute? To me it was always obvious that PI3K was going to be a major driver in the Warburg effect, having realized that virtually everything insulin does, including driving glucose uptake, is regulated through the PI3K pathway. The Warburg effect can be generated independently of PI3K – but the discovery of the kinase provided a mechanistic basis for the effect. The question that intrigued me is what advantage is conferred to cancer cells by a higher rate of glycolysis under conditions where they could synthesize ATP more efficiently through oxidative phosphorylation – this seems counterintuitive.

Matt Vander Heiden, Craig Thompson and I recently put forward a model based on the realization that making ATP is not that big a problem for cancer cells. I had discussions with Efraim Racker (who had overlapped with Warburg) way back in the 70s, in which he pointed out that cancer cells actually make too much ATP. High levels of ATP can ‘back up’ in mitochondria and trigger several problems, including production of reactive oxygen species (ROS) and inhibition of glycolysis. Efraim and others were looking at ways that cancer cells get rid of excess ATP rather than wondering how they can make enough. This stimulated me to consider that the increased uptake of glucose in cancer cells might confer an advantage in terms of generating biomass, rather than generating ATP.

The increase in aerobic glycolysis can be seen as being akin to building a town beside a large river as opposed to building it besides a little stream. Maybe the little stream would have enough water to keep everybody hydrated in the town, but wouldn't it be nice to have so much excess water coming by that you can grab as much as you want, any time, to fulfil any need? So, let's say that the cancer cell suddenly needs to make NADPH; it can just grab at some of the glucose that is flowing by and send it down the pentose phosphate shunt. Suddenly it needs to make more ribose – it can do this through the non-oxidative pathway, if NADPH isn't needed. Maybe 90% of the time the extra carbon atoms aren't needed at all, so they can just be spat out as lactate. But in order to meet the acute demands that a cell will periodically have, like going through the cell cycle, having a whole flood of carbon atoms on hand to generate building blocks such as alanine, serine and glycine instantly, provides a real advantage. The other thing to keep in mind is that if the cell makes most of its ATP through glycolysis, then the mitochondrion can spend its time performing macromolecular synthesis rather than oxidative phosphorylation. This is essentially why I think that most tumors do glycolysis at a higher rate.

“In (Warburg's) days, everyone treated cancer as though it is a single disease, so phenomena that weren't observed across all cancer types were considered to be irrelevant to its etiology”

**Another controversial aspect of your research is the use of vitamin C to treat cancer. What mechanism is involved and how likely is it that vitamin C will one day be used as an anti-cancer therapy?**

I am very optimistic about the potential of vitamin C in cancer therapy. The fact that oncologists have been reporting positive responses for years, even without any concrete hypothesis and without proper selection of patients, is promising. And I'm not referring to alternative medicine clinics, where patients are usually given oral vitamin C at levels that will never reach a high enough dose to kill the tumors. Serious oncologists at cancer centers worldwide have been successfully using vitamin C as a co-therapy with chemotherapeutic drugs for years. There have clearly been enough responders to keep people interested, despite conflicting results. The challenges are to pinpoint the patients who will benefit most, to figure out the right dose to administer and to determine if combinations will work better than vitamin C alone, and for this, we need to understand the exact mechanism by which it can kill tumor cells.

We have recently shown that high levels of vitamin C can kill aggressive colorectal cancer cells with *KRAS* or *BRAF* mutations, and that the mechanism involves uptake of the oxidized form of vitamin C, dehydroascorbic acid (DHA). This triggers oxidative stress and cell death. For this to work, the tumor cells need to be generating high levels of ROS – which are spat out of the cell into the plasma, where they oxidize vitamin C – and to have high expression of the glucose transporter GLUT1 that is needed for uptake of DHA. Both *KRAS*-mutant and *BRAF*-mutant colorectal cancers have these characteristics, and it is likely that other tumors will similarly be responsive to vitamin C. Other drugs or treatments that increase ROS or expression of GLUT1 might work synergistically with vitamin C. For example, radiation, which induces ROS, might work more effectively in the presence of high levels of vitamin C. So, there is a clear logic now as to how you would select patients for the clinical trial, but we need to follow patient responses closely to make sure our predictions are accurate and that we don't miss anything.

There are two things that worry me about existing clinical trials using vitamin C. The first is that these trials often combine another reducing agent, an antioxidant, with vitamin C. We have shown in mice that if we add reducing agents such as *N*-acetylcysteine, vitamin C doesn't work anymore. The two completely cancel each other out. The other thing to consider is that if you have high serum levels of glucose, it is going to impair the ability of the tumor cells to take up DHA because glucose and DHA enter the cell through the same transporter. So it's important to avoid giving something that will raise serum glucose together with vitamin C therapy. A lot of oncologists in the US give patients a nutritional drink such as Ensure® during therapy, and because of the high sugar content, this makes serum glucose levels very high. Oncologists need to realize that there are a number of things that can compromise the response to vitamin C therapy.

**How much is known about the potential toxicity of vitamin C to normal cells when administered at high doses?**

Obviously there are a lot of cells in your body that have high rates of glucose uptake, including your brain and muscles. If serum ascorbate were converted to DHA in these tissues, they would take it up and this could cause oxidative damage. However, most normal tissues do not produce the high levels of ROS needed to convert ascorbate to DHA. Also, if DHA is taken up by normal cells, they have a sufficient reducing potential to deal with it better than tumor cells. Toxicity trials have already been done, and people have tolerated more than 10 mM of ascorbic acid in the serum over long periods of time without any obvious side effects; compared to chemotherapy, the adverse effects are minimal. The tumor is selectively vulnerable, which makes this an attractive therapeutic strategy.

**Does that mean that high levels of dietary vitamin C could protect against cancer?**

You can't eat enough vitamin C to really have much effect – the amount we used is equivalent to that in about 300 oranges. It's also got to be given intravenously because oral doses are not efficiently absorbed to give high serum levels. For now, the therapy needs to be tested in a clinical trial setting where all the variables that I've discussed can be properly monitored.

**Moving on to your career path, what motivated you to come back to Cornell in 2012?**

I think it was the opportunity to build a cancer center in an environment where basic scientists and clinicians were really interested in working together. One of the biggest challenges we have at most institutions, particularly in large cancer centers, is that people tend to fall into high specialty areas and don't communicate with specialists outside of their areas, in part because of differences in vocabulary. When clinicians start talking about what they are doing in the clinic and basic scientists don't understand a lot of the terminology, and vice versa, communication breaks down. I was looking for a place that was about the right size to allow me to build better communications. Resources and infrastructure are needed to do this, and I knew that New York City was well equipped. My goal was to create a team of people working from bench to bedside and bedside back to bench who could interact and had all the resources to make things happen. At this point in my career I wanted to be able to see things that I had discovered – like vitamin C in cancer, and PI3K inhibitors – get converted into new therapies to benefit patients.

“My goal was to create a team of people working from bench to bedside and bedside back to bench who could interact and had all the resources to make things happen”

**Tell us a little bit about your goals in the ‘Stand Up to Cancer’ initiative. Why are ovarian and breast cancers so difficult to treat?**

With regard to breast cancers, much progress has been made in therapies for estrogen-receptor (ER)- and/or progesterone-receptor (PR)-positive breast cancers, and for HER2-positive breast cancers, through development of drugs that target the events driving the growth of these tumors: blocking estrogen production or directly targeting the ER, or directly targeting HER2. However, breast cancers that are classed as being triple negative – defined by the observation that such cancers do not have high levels of ER, PR or HER2 – don't respond to these drugs. They do respond to chemotherapy and radiation but almost invariably re-emerge after therapy. Sequencing of DNA and RNA from breast cancer cells reveals that the triple-negative breast cancers typically have defects in DNA repair genes, including germline or sporadic defects in the DNA repair genes *BRCA1* or *BRCA2*. Because of these DNA repair defects, these tumors have extensive chromosomal rearrangements that result in deletions of tumor suppressors such as *PTEN*. By contrast, the ER- and/or PR-positive, and HER2-positive, tumors often have point mutations in *PIK3CA,* the gene encoding PI3K. Loss of *PTEN* or mutations in *PIK3CA* both result in hyperactivation of the PI3K pathway, raising the possibility that these inhibitors could be useful for treating all subtypes of breast cancers.

Surprisingly, a comparison of genetic aberrations revealed that triple-negative breast cancer is more similar to ovarian cancer than to other subtypes of breast cancer, suggesting that these two types of tumor might respond to the same therapies. Because triple-negative breast cancers and ovarian cancers often have defects in DNA repair, we can reason that inhibiting a back-up pathway for DNA repair involving PARP could result in catastrophic DNA damage and cell death in these tumors. Indeed, the PARP inhibitor olaparib has recently been approved for treating ovarian cancers with mutations in BRCA genes. Our ‘Stand Up to Cancer’ team developed clinical trials to determine whether combining PI3K inhibitors with other targeted therapies for breast cancers enhances responses and prolongs life. The results look very promising, in particular when PI3K inhibitors are combined with anti-estrogen therapy in ER-positive breast cancers, and when PI3K inhibitors are combined with PARP inhibitors in triple-negative breast cancers and ovarian cancers. Hopefully, these novel combinations of targeted therapies will cause more durable responses and prevent relapses.

**How important have your many collaborations and interactions with others been in guiding your research?**

I've always felt that there isn't a need to reinvent the wheel. If you know somebody who uses a different technology and has the kind of expertise you're looking for, it is always best to collaborate with them first, and then if the technology turns out to be really useful you can import it into your own lab. Most of my publications have ten or so authors and involve two or three labs. I always like to see everything reproduced separately in other labs – if the same result is obtained independently in a different environment, you can have confidence in it. Also, almost all our papers have both an *in vivo* and *in vitro* component to them. We usually tease something out *in vitro* first, using cell lines, and then go to genetically engineered mice to try to reproduce the result in an *in vivo* setting. This requires that we have a broad range of approaches and experts available to us.

I stipulate to my post-docs and graduate students that they have to be able to understand in great detail how each part of their data was obtained, even if it was done in somebody else's lab. They have to be involved in experiments performed in collaboration with others, so that the work can be reproduced. This also helps in being able to import new and important techniques back into the lab, so that you can keep moving forward competitively in the field.

**You are the co-founder of two biotechnology companies, Agios and Petra. Do you think that more academic scientists need to have a foot in industry?**

I have started several companies over the years. Back in the 80s, I was involved in starting a company that is now called Advance Magnetics and is still going, and I started another company in the 90s that failed because of finances. Agios, however, has been very successful, having brought multiple drugs to the clinic in a very short time. I'm hopeful that Petra will follow a similar course. As I said, at this stage in my life, I want to see discoveries translated into drugs. For some discoveries – such as vitamin C – you don't need to start a company because the agent already exists and it's cheap and easy to use. We are good at ‘repurposing’ drugs and using combinations of drugs in academia, but significant investment is needed to drive development of new drugs from discovery through to clinical trials, and very few academic centers have sufficient resources. It's easier to do this in a pharma setting where venture capital money can fund the pipeline. The alternative for an academic is to collaborate with industry, and I've done that too. Many pharma companies are now set up to harvest the research of academic institutions and to work hand-in-hand with them to properly design clinical trials. Academics are good at figuring out what the biomarkers should be and how to design the trials but not quite so good at medicinal chemistry and optimizing the pharmacokinetics and pharmacodynamics. Working together with pharma is crucial to make this happen in a rapid way – speed is of the essence in drug development. We're at a time when we have the opportunity to understand cancer at the molecular level and develop therapies in a scientific manner, rather than empirically.

“We're at a time when we have the opportunity to understand cancer at the molecular level and develop therapies in a scientific manner, rather than empirically”

**What key advice would you give to an early-stage researcher?**

I wouldn't say everyone should follow in my footsteps – there are many paths to success. But what has particularly benefited me throughout my career is my understanding of biochemistry and chemistry. Unless I understand something at the chemical or biochemical level, I don't feel like I really understand it. I am always driven to understand a phenomenon I see in molecular detail so there is no question about how it works. A lot of people would find this boring, but to me this approach has always led to unexpected results and breakthroughs. When you think you know how something works but then get down to the minute detail and realize that you actually don't, this often opens the door to a new discovery – almost all my successes have arisen this way. I have to admit that luck has been important too. For example, impurities in commercial reagents have led to many of my discoveries. When you see an unexpected result that is due to an impurity in a reagent, if that unexpected result looks really interesting, it might be worth figuring out what the impurity is. A desire to understand how things work at the molecular level has allowed me to take advantage of what other people would toss away. That kind of dogged search is what has led me to new discoveries.

“A desire to understand how things work at the molecular level has allowed me to take advantage of what other people would toss away. That kind of dogged search is what has led me to new discoveries”

**If you hadn't chosen science, what would you be doing now?**

I would probably be an architect or maybe a gardener, although both of these professions are really science driven.

**What do you enjoy doing outside of the lab?**

Reading, eating great food (no dessert or added sugar!) and drinking great wine (dry rather than sweet), with family and friends and, when possible, snorkeling.

